# Age at *Mycobacterium bovis* BCG Priming Has Limited Impact on Anti-Tuberculosis Immunity Boosted by Respiratory Mucosal AdHu5Ag85A Immunization in a Murine Model

**DOI:** 10.1371/journal.pone.0131175

**Published:** 2015-06-22

**Authors:** Daniela Damjanovic, Amandeep Khera, Sam Afkhami, Rocky Lai, Anna Zganiacz, Mangalakumari Jeyanathan, Zhou Xing

**Affiliations:** McMaster Immunology Research Centre and Department of Pathology & Molecular Medicine, McMaster University, Hamilton, Ontario, Canada; University of Delhi, INDIA

## Abstract

Tuberculosis (TB) remains a global pandemic despite the use of Bacillus Calmette-Guérin (BCG) vaccine, partly because BCG fails to effectively control adult pulmonary TB. The introduction of novel boost vaccines such as the human Adenovirus 5-vectored AdHu5Ag85A could improve and prolong the protective immunity of BCG immunization. Age at which BCG immunization is implemented varies greatly worldwide, and research is ongoing to discover the optimal stage during childhood to administer the vaccine, as well as when to boost the immune response with potential novel vaccines. Using a murine model of subcutaneous BCG immunization followed by intranasal AdHu5Ag85A boosting, we investigated the impact of age at BCG immunization on protective efficacy of BCG prime and AdHu5Ag85A boost immunization-mediated protection. Our results showed that age at parenteral BCG priming has limited impact on the efficacy of BCG prime-AdHu5Ag85A respiratory mucosal boost immunization-enhanced protection. However, when BCG immunization was delayed until the maturity of the immune system, longer sustained memory T cells were generated and resulted in enhanced boosting effect on T cells of AdHu5Ag85A respiratory mucosal immunization. Our findings hold implications for the design of new TB immunization protocols for humans.

## Introduction

The unrelenting nature of the global tuberculosis (TB) epidemic is partly due to the inefficacy of BCG, the only licensed vaccine against TB, as it fails to effectively control adult pulmonary TB in endemic countries [1, 2]. This emphasizes an urgent need for an effective novel boost vaccine, which could improve and prolong the protective immunity of BCG-induced immunity [3–5]. In this regard the human Adenovirus 5-vectored TB vaccine (AdHu5Ag85A) developed in our laboratory has been shown to effectively boost BCG in a variety of animal models when administered via the respiratory mucosal route [6–10]. Thus, it is among the major candidate vaccines currently considered to be introduced into the human immunization program for respiratory mucosal boost immunization in BCG vaccinees [7–11]. Notably, an advanced candidate TB vaccine, modified vaccinia virus Ankara (MVA) expressing Ag85A has already been evaluated in healthy humans for aerosol vaccination and found to be immunogenic and safe [12].

BCG immunization is given via the intradermal route to most of the world’s newborns, while some countries administer it to infants or school age children [4, 13]. The optimal age for BCG immunization still remains controversial, as seen in the divergent ages implemented in different countries. As children under five years of age are most at risk, BCG needs to be administered during the first five years of life [14, 15]. However, early life immunization is threatened by an under-developed immune system in this population [16–18]. Results from ongoing studies as to whether age at BCG vaccination differentially impacts BCG-induced anti-TB are inconclusive [19–21]. Considering the future implementation of boost vaccination in BCG vaccinees, knowledge of optimal timing for BCG priming and for subsequent boost vaccination is critical in order to implement successful vaccination strategies.

Neonatal murine models have been extensively used to study the human infant responses to infections because of the similarities in postnatal immune maturation in humans and mice [16, 22–24]. However, the dynamics of immune maturity are distinct in humans and mice. Immune cell functions mature in neonatal mice through the weaning period, which is the first three weeks of life. However, the human neonatal immune system is more mature at birth but takes two years to reach complete maturity [22, 23, 25]. Thus, 2–3 week old infant mice and 6–8 week old adult mice well represent the immature and the mature immune systems in humans, respectively, and provide excellent modeling of BCG priming and subsequent boost AdHu5Ag85A immunization to inform us of the potential optimal timing for BCG priming and subsequent boosting in humans.

The purpose of this study was to determine whether protection against pulmonary tuberculosis in mice primed parenterally with BCG and subsequently boosted by intranasal AdHu5Ag85A immunization is dependent on the age at which mice were vaccinated with BCG, and also to evaluate the effect of elapsed time between BCG prime and AdHu5Ag85A boost immunization on T cell immunity. Our data show that despite differences in T cell responses, the age at BCG immunization had a limited effect on the protective anti-TB immunity induced by BCG immunization and subsequent boost AdHu5Ag85A respiratory mucosal immunization. Furthermore, while longer elapsed time between BCG priming and AdHu5Ag85A boosting led to reduced AdHu5Ag85A boosting effect in mice BCG immunized as infants, it led to enhanced AdHu5Ag85A boosting effect in mice BCG immunized as adults. Our findings will help develop immunization protocols involving the use of BCG and virus-based boost vaccines in humans.

## Materials and Methods

### Animals for *in vivo* models

Breeding pairs of BALB/c mice (6–8 weeks old) were purchased from Charles River Laboratories (Saint-Constant, Quebec, Canada) and bred in a specific pathogen free level B facility at McMaster University. Both male and female offspring were used in the experiments. Mice at the time of weaning (3 weeks old) were considered as infants and 8-week-old mice were considered as adults. All experiments were conducted in accordance with and approval from the animal research ethics board of McMaster University.

### Parenteral BCG priming and respiratory mucosal AdHu5Ag85A boosting immunization

Parenteral BCG immunization was carried out by subcutaneous (s.c.) delivery over the flank, of 5 x 10^4^ colony forming units (cfu) to infant or 1 x 10^5^ cfu to adult mice in 100 μl (50 μl on each side of flank) of BCG Pasteur [26]. Different doses were chosen for infant and adult mice to reflect the general practice in humans where half the adult dose is administered to children under the age of 12 years [27]. BCG Pasteur bacilli were grown in Middlebrook 7H9 broth supplemented with Middlebrook oleic acid-albumin-dextrose-catalase enrichment, 0.002% glycerol, and 0.05% Tween 80 for 10–15 days, aliquoted and stored in -70°C until use. Before each *in vivo* use, BCG bacilli were washed with PBS containing 0.05% Tween 80 twice and passed through a 27-gauge needle 10 times to disperse clumps. Boost AdHu5Ag85A immunization was carried out via intranasal route with 5 x 10^7^ plaque forming units (pfu) in 25 μl [7, 26, 28].

### *Mycobacterium tuberculosis* preparation and animal models of pulmonary tuberculosis

*M*.*tb* H37Rv bacilli were grown in Middlebrook 7H9 broth supplemented with oleic acid-albumin-dextrose-catalase enrichment, 0.002% glycerol, and 0.05% Tween 80 was used to grow for 10–15 days, aliquoted and stored in -70°C until use. Before each *in vivo* use, *M*.*tb* bacilli were washed with PBS containing 0.05% Tween 80 twice and passed through a 27-gauge needle 10 times to disperse clumps. Pulmonary infection with *M*.*tb* H37Rv was carried out as previously described [6, 7, 26, 28]. The levels of mycobacterial infection in the lung and spleen were determined by colony forming assay, by plating serial dilutions of tissue homogenates in triplicates onto Middlebrook 7H10 agar plates. Plates were incubated at 37°C for 15–17 days before colony enumeration.

### Bronchoalveolar lavage and lung mononuclear cell isolation

Mice were euthanized by exsanguination under anaesthesia by gaseous isoflurane, in accordance with the animal research ethics board of McMaster University. Mononuclear cells from bronchoalveolar lavage (BAL), lung and spleen were isolated as previously described [7, 28]. Subsequently, lungs were perfused through the left ventricle with Hank’s buffer to remove leukocytes from the pulmonary vasculature. Briefly, the lungs were cut into small pieces and incubated in 10 ml/lung of 150 U/ml collagenase type I (Sigma-Aldrich, St. Louis, MO) for 1 hour at 37°C, after which the lung pieces were crushed through 40-μm basket filters. To isolate mononuclear cells, spleens were crushed using the frosted ends of sterile glass slides, and filtered through 40-μm basket filters. Red blood cells in the lungs and spleens were lysed with ACK lysis buffer, and cell pellets resuspended in complete RPMI medium (RPMI 1640 supplemented with 10% FBS, 1% penicillin-streptomycin, and 1% L-glutamine).

### Cell stimulation, tetramer staining, intracellular cytokine staining, and flow cytometry

Mononuclear cells from BAL, lungs, or spleens were cultured in U-bottom 96-well plates at a concentration of 20 million cells/ml for lungs and spleens and 0.5 million cells/ml for BAL. Tetramer staining was performed on unstimulated cells using a PE-conjugated tetramer for the immunodominant Ag85A CD8 T cell peptide MPVGGQSSF on the BALB/c MHC class I allele H-2L^d^ (MHC Tetramer Laboratory of Baylor College of Medicine, Houston, TX). For intracellular cytokine staining, mononuclear cells were stimulated with crude BCG (cBCG) and *M*.*tb* culture filtrate (CF) at a concentration of 1 μg/well for 24h with the last 6h in the presence of Golgi plug (5 mg/ml Brefeldin A; BD Pharmingen, San Jose, CA), or left unstimulated. The cBCG was prepared by us by UV inactivation, and the CF was purchased from bei Resources (Manassas, VA). A mixture of cBCG and CF was used, as in our previous experience this is an optimal stimulation that reflects immune activation. In selected experiments, cells were cultured in the presence of Golgi plug with or without stimulation for 6h with an Ag85A-specific CD8 peptide (MPVGGQSSF) or Ag85A-specific CD4 peptide (LTSELPGWLQANRHVKPTGS) at a concentration of 2.5 μg/well. After incubation, cells were washed and blocked with CD16/CD32 (BD Biosciences, San Jose, CA) in 0.5% bovine serum albumin-PBS for 15 minutes on ice. Subsequently, cells were stained for extracellular markers and then fixed and permeabilized according to manufacturer’s instructions (BD Biosciences, San Jose, CA) for the detection of intracellular cytokines. The antibodies used included V450-anti-CD3, PECy7-anti-CD4, APC-Cy7-anti-CD8, APC-anti-IFN-γ, FITC-anti-TNF-α, and PE-anti-IL-2 (BD Biosciences, San Jose, CA). In a set of experiments unstimulated cells were stained for memory T cell markers (APC-Cy7-anti-CD4, APC-anti-CD8, V500-anti-CD44) (BD Biosciences, San Jose, CA), and (PECy7-anti-CD62L, FITC-anti-CD127) (eBioscience, San Diego, CA). Stained cells were collected on the LSRII flow cytometer (BD Biosciences, San Jose, CA) and analyzed using FlowJo software (Tree Star, Inc., Ashland, OR). Cells were assessed as total number of cells per tissue compartment by multiplying the frequency of total of the population of interest by total cell number in the tissue.

### Pulmonary histopathological analysis

Lungs were kept in 5 ml of 10% formalin for at least 72h before embedding in paraffin. Tissue sections were stained with hematoxylin and eosin (H&E) for histological examination at 5x magnification.

### Statistical analysis

To determine if the differences among groups were significant, Student’s *t* test was performed for two-sample comparisons with Prism (GraphPad Software, Inc., La Jolla, CA). A *p* value of <0.05 was regarded as statistically significant.

## Results

### Mice BCG-immunized as infants mount a comparable magnitude of antigen-specific CD4^+^IFN-γ^+^ T cell responses with varying kinetics compared to mice immunized as adults

**Fig 1:**
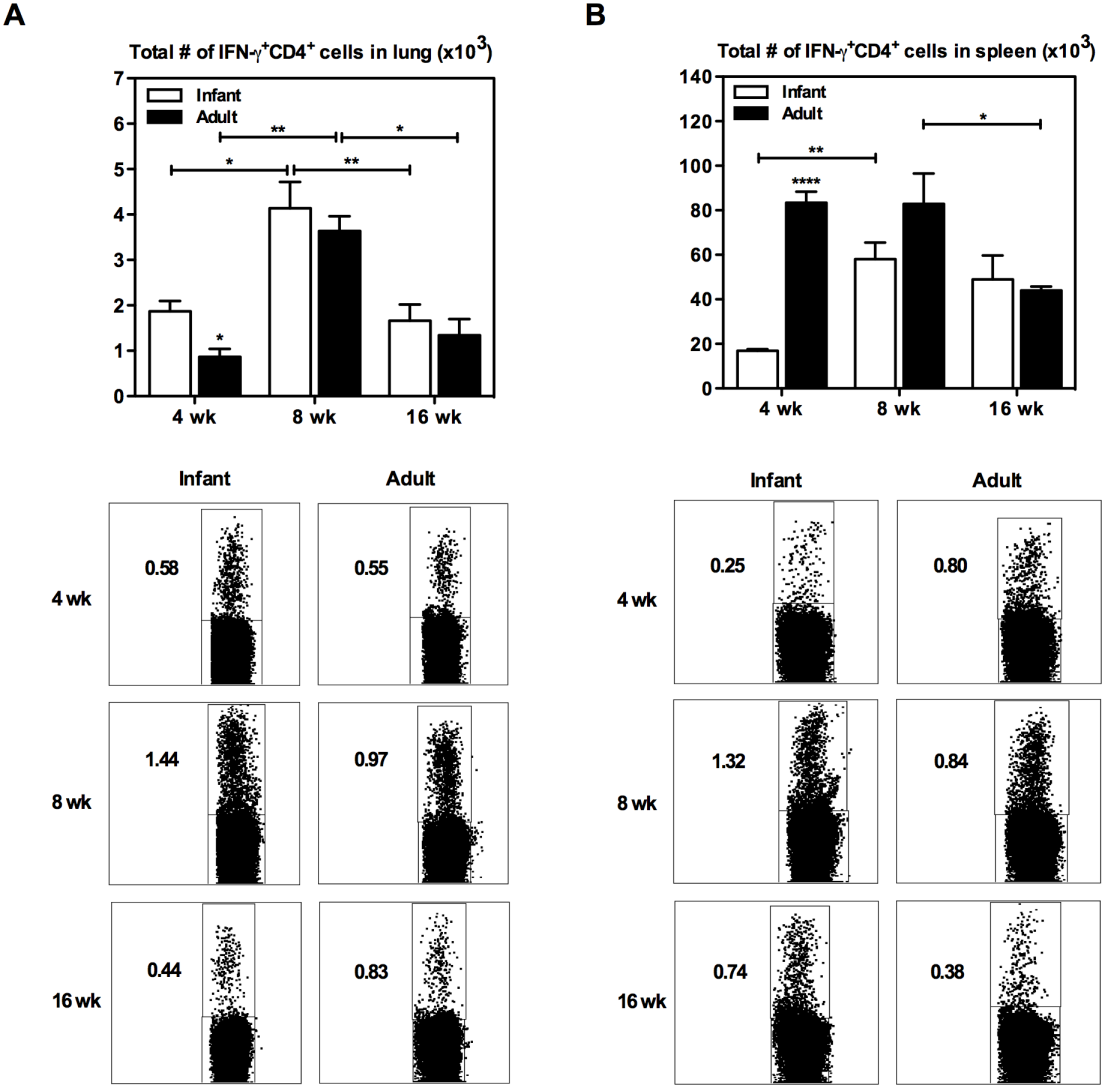
IFN-γ^+^CD4^+^ T cell kinetics in the lung and spleen of infant and adult mice following BCG immunization. Infant and adult mice were immunized s.c. with BCG and sacrificed at 4, 8, or 16 weeks following immunization. Cells from the lung (A) and spleen (B) were isolated and stimulated with mixed *M*.*tb* culture filtrate (CF) and crude BCG (cBCG) for 24h or with media only as a control (unstimulated). Cells were stained with extracellular cell markers, followed by intracellular staining for IFN-γ, and analyzed by flow cytometry. Absolute numbers of IFN-γ^+^CD4^+^ T cells in the tissues were calculated (unstimulated numbers were subtracted from stimulated), and representative dot plots are shown. Results are from one to two independent experiments per timepoint, n = 4-8/group/timepoint. Data are expressed as Mean ± SEM. *, p < 0.05; **, p < 0.005; ****, p < 0.0001.

Parenteral BCG immunization in adult mice leads to induction of Th1 responses [26, 29]. To begin evaluating the impact of age at BCG immunization on BCG-induced immunity, BALB/c mice were either immunized at 3 weeks of age (infant) or 8 weeks of age (adult). At specified time points post-immunization Ag-specific IFN-γ^+^CD4^+^ T cell responses were enumerated by flow cytometry after *in vitro* stimulation of lung and spleen mononuclear cells with crude mycobacterial antigens (CF+cBCG). Analysis of longitudinal kinetics of Ag-specific responses in the lung revealed that the total number and frequency of Ag-specific IFN-γ^+^CD4^+^ T cell responses peaked at 8 weeks and contracted significantly by 16 weeks post-immunization in both groups of mice (Fig 1A). Although BCG immunization in infant and adult mice led to a comparable magnitude of Ag-specific responses at 8 and 16 weeks post-immunization, a significantly increased number of IFN-γ^+^CD4^+^ cells was found in the lung at 4 weeks in mice BCG immunized as infants compared to mice immunized as adults (Fig 1A). Unlike in the lung, longitudinal kinetics of Ag-specific responses in the spleen varied between the two groups. While Ag-specific responses in the spleens of mice immunized as infants peaked at 8 weeks post-immunization, responses in mice immunized as adults reached the highest magnitude by 4 weeks (Fig 1B). Of note, although over time in mice immunized as adults the Ag-specific responses significantly contracted by 16 weeks post-immunization, in mice immunized as infants these responses did not contract by 16 weeks (Fig 1B). Furthermore, in contrast to the lung a significantly increased number of IFN-γ^+^CD4^+^ cells was found in the spleen at 4 weeks post-immunization in mice BCG immunized as adults compared to those immunized as infants (Fig 1B). The above data suggest that regardless of age at BCG immunization a strong Th1 response is generated.

### Mice BCG-immunized as infants elicit antigen-specific CD4 T cell responses with similar multi-cytokine functionality as mice immunized as adults

**Fig 2:**
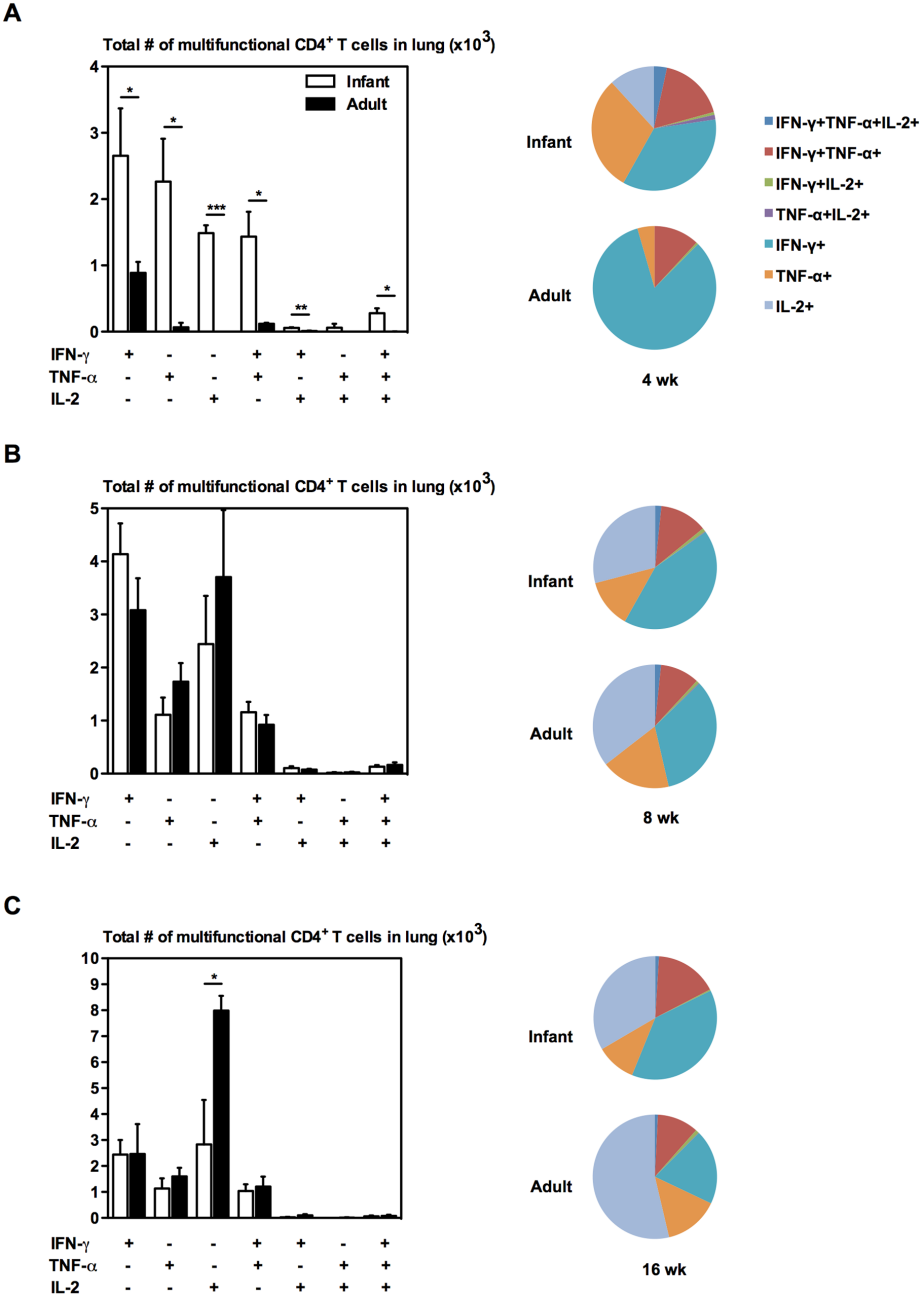
Multifunctional profile of CD4^+^ T cells in the lung of infant and adult mice following BCG immunization. Infant and adult mice were immunized s.c. with BCG and sacrificed at 4 (A), 8 (B), or 16 (C) weeks following immunization. Cells from the lung were stimulated with *M*.*tb* CF + crude BCG for 24h or left unstimulated as a control. Cells were stained and analyzed by flow cytometry. Average proportions displayed in pie chart are of the CD4 T cells expressing specific cytokine combinations. Absolute numbers of CD4^+^ T cells in the tissues were calculated and displayed in bar graphs. Results are from one independent experiment per timepoint, n = 4-5/group/timepoint. Data are expressed as Mean ± SEM. *, p < 0.05; **, p < 0.005; ***, p < 0.0005.

Since deficient T cell multifunctionality has been reported among infants, primarily in the CD4 T cell compartment [30, 31], next we investigated the multifunctional profile among Ag-specific T cells. Generally, Ag-specific CD4 T cells induced by BCG immunization were positive for a single cytokine regardless of age at immunization (Fig 2). Only a small proportion of Ag-specific CD4 T cells were multifunctional in each group, which remained unchanged throughout the post-immunization period (Fig 2A–2C). Detailed analysis of multi-cytokine expression combinations at 4 weeks post-immunization revealed almost equal proportions of cells that express either IFN-γ or TNF-α or IL-2 in mice immunized as infants, as opposed to predominantly IFN-γ-expressing cells in mice immunized as adults (Fig 2A). Furthermore, mice immunized as infants had significantly increased numbers of IFN-γ, TNF-α, and IL-2 single-cytokine-positive cells and IFN-γ+TNF-α+ cells, compared to mice immunized as adults (Fig 2A). However, multi-cytokine functionality was comparable at 16 weeks post-immunization except for IL-2 single-cytokine-positive cells (Fig 2C). The proportion of IL-2-producing Ag-specific CD4 T cells was significantly increased in mice immunized as adults (Fig 2C). The above data suggest that although there were transient differences in the multi-cytokine functionality of Ag-specific CD4 T cells, such variations became minimal later after immunization.

### Mice BCG-immunized as adults but not as infants generate longer sustained memory T cells

**Fig 3:**
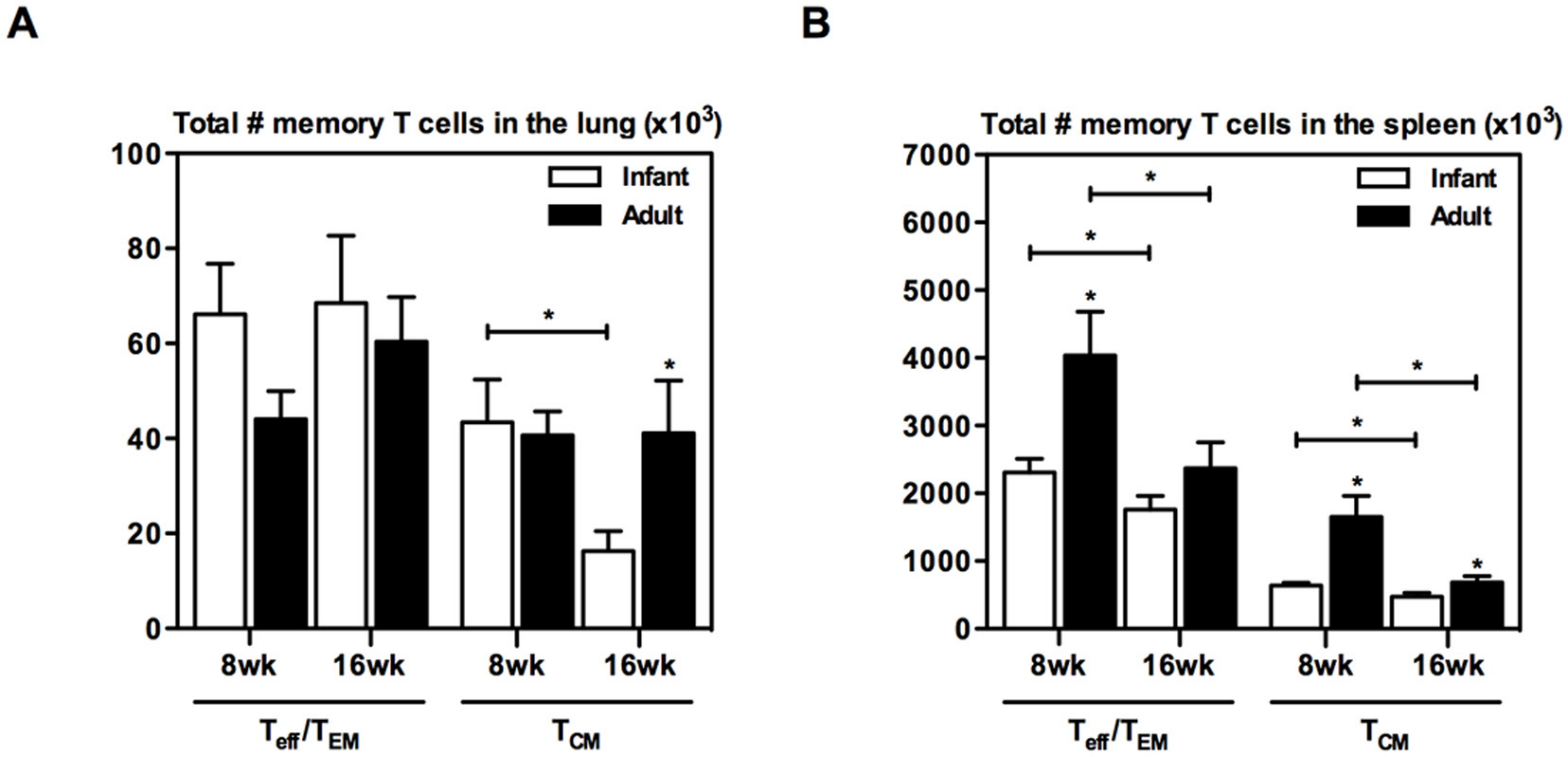
Memory T cells in the lung and spleen of infant and adult mice following BCG immunization. Infant and adult mice were BCG immunized and sacrificed at 8 or 16 weeks. Cells from the lung (A) and spleen (B) were stained with extracellular antibodies for memory CD4 T cell markers and analyzed by flow cytometry. Absolute numbers of CD4^+^CD44^+^ cells that are T_eff_/T_EM_ (CD127^-/+^CD62L^-^) or T_CM_ (CD127^+^CD62L^+^) in the tissues were calculated. Results are from one experiment per timepoint, n = 4/group/timepoint. Data are expressed as Mean ± SEM. *, p < 0.05.

Having established that age at BCG immunization does not affect the magnitude and functionality of antigen-specific T cells and that IL-2-producing antigen-specific CD4 T cells are generated in both groups, we next evaluated the memory phenotype of these cells. Of note, BCG vaccine-activated IL-2 expression has been previously shown to be associated with long-lived memory T cells with a central memory phenotype [32]. To differentiate between cells having the phenotype of T_eff_ (effector), T_EM_ (effector memory) and T_CM_ (central memory), we evaluated the expression of multiple surface markers including CD44 (to identify activated T cells), CD62L, and CD127 [29]. In the lungs, levels of T_eff_ /T_EM_ (CD44^+^CD62L^-^CD127^+/-^) were similar between mice BCG immunized as infants and mice BCG immunized as adults at 8 and 16 weeks post-immunization (Fig 3A). Similarly, levels of central memory T cells (T_CM_) (CD44^+^CD62L^+^CD127^+^) were comparable between the two groups at 8 weeks (Fig 3A). In contrast, at 16 weeks post-immunization mice BCG immunized as adults had significantly higher levels of central memory T cells compared to mice immunized as infants (Fig 3A). Furthermore, while T_CM_ cells significantly decreased in number overtime in mice BCG immunized as infants, they remained sustained in mice immunized as adults (Fig 3A). In contrast to the lung, levels of T_eff_ /T_EM_ cells were significantly higher in the spleen of mice BCG immunized as adults compared to mice immunized as infants at 8 weeks post-immunization (Fig 3B). However, such differences faded by 16 weeks (Fig 3B). In the spleen, a significantly increased number of T_CM_ cells was observed in mice BCG immunized as adults compared to mice immunized as infants throughout the study period (Fig 3B). However, in both groups T_CM_ cell numbers significantly decreased over time (Fig 3B). Taken together the above data suggest that BCG immunization in adult mice leads to generation of higher and sustained levels of central memory cells in the spleen compared to BCG immunization in infant mice.

### Respiratory mucosal AdHu5Ag85A boost immunization leads to increased antigen-specific responses in mice BCG-immunized as adults compared to mice immunized as infants

**Fig 4:**
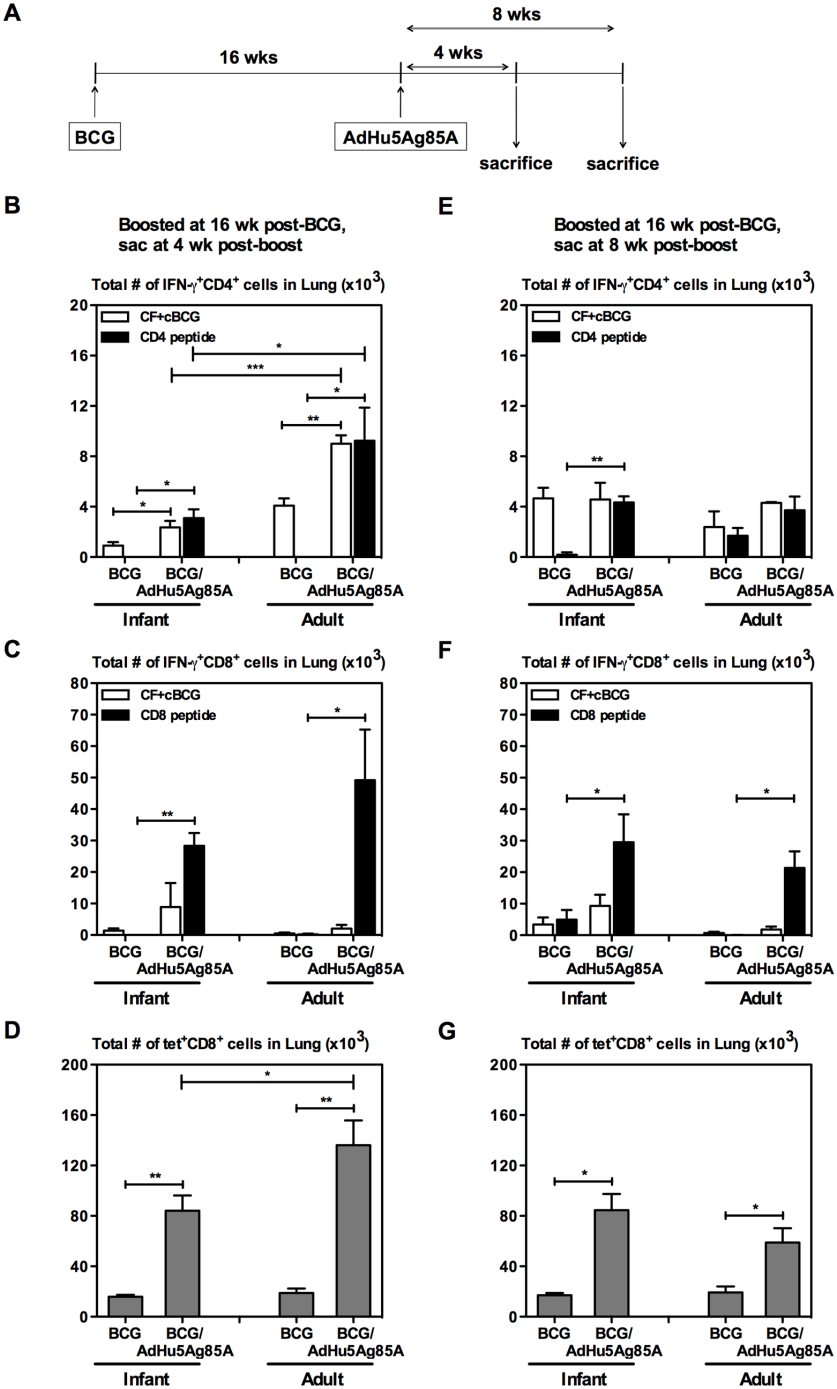
Ag-specific T cell responses in the lung after boost AdHu5Ag85A immunization in BCG primed mice. Infant and adult mice were BCG immunized, and the AdHu5Ag85A booster vaccine was administered i.n. at 16 weeks post-BCG (A). The mice were sacrificed 4 weeks (B, C, D) or 8 weeks (E, F, G) after boosting. Lung cells were stimulated either with *M*.*tb* CF + crude BCG for 24h (open bar), or Ag85A-specific CD4 or CD8 T cell peptide for 6h (black bar), or left unstimulated (B, C, E, F). Cells were stained and analyzed by flow cytometry. Absolute numbers of IFN-γ^+^CD4^+^ (B, E) and IFN-γ^+^CD8^+^ (C, F) T cells were calculated (unstimulated subtracted from stimulated). Ag85A CD8 peptide tetramer staining was performed on lung cells, and analyzed by flow cytometry (D, G). Absolute numbers of tet^+^CD8^+^ T cells were calculated. Results are from one experiment per timepoint, n = 4-5/group/timepoint. Data are expressed as Mean ± SEM. *, p < 0.05; **, p < 0.005; ***, p < 0.0005. All other comparisons (not indicated) were not significant.

Having systematically investigated the nature of immunogenicity following parenteral BCG immunization at different ages as described above, we next set out to examine the effect on AdHu5Ag85A boost immunization. To this end, a group of infant and a group of adult mice were immunized via the parenteral route with BCG (Fig 4A). At 16 weeks post-BCG a set of mice immunized as infants or adults were boosted with AdHu5Ag85A via the intranasal route (BCG/AdHu5Ag85A) (Fig 4A). Another set of mice was left without boosting as controls (BCG). At 4 or 8 weeks post-boost mice were sacrificed and antigen-specific responses in the bronchoalveolar lavage (BAL) and lung were enumerated (Fig 4A). BAL cells were *in vitro* stimulated with crude-mycobacterial antigens (CF+cBCG) and lung mononuclear cells were stimulated with either CF+cBCG or Ag85A CD4 or CD8 peptides and analyzed for Ag-specific IFN-γ^+^CD4^+^ and IFN-γ^+^CD8^+^ responses. In addition, lung mononuclear cells were stained for Ag85A CD8 tetramer. Consistent with our previous findings [26, 29] parenteral BCG priming alone did not elicit airway luminal CD4 or CD8 T cell responses in either group (data not shown). On the other hand, intranasal AdHu5Ag85A boosting markedly increased multi-mycobacterial antigen-specific CD4 and CD8 T cells in the airway lumen in both groups at 4 weeks post-boost (Table 1). Although Ag-specific responses contracted, they were sustained in the BAL in both groups at 8 weeks post-boost (Table 1). BCG priming alone induced comparable levels of multi-mycobacterial antigen (CF+cBCG) reactive CD4 (Fig 4B and 4E) and CD8 (Fig 4C and 4F) T cells in the lungs of both groups. In comparison, as expected intranasal AdHu5Ag85A boost significantly increased multi-mycobacterial antigen-specific and Ag85A-specific CD4 T cells (Fig 4B) and Ag85A-specific CD8 T cells (Fig 4C) in the lungs of both groups at 4 weeks post-boost. However, intranasal AdHu5Ag85A boosting in mice BCG immunized as adults resulted in significantly increased multi-mycobacterial antigen-specific CD4 T cells and Ag85A-specific CD4 T cells in the lung compared to mice BCG immunized as infants at 4 weeks post-boost (Fig 4B). Furthermore, at 4 weeks post-boost although boosting enhanced the Ag85A-specific tetramer+ CD8 T cells in both groups of mice, the extent of enhancement was greater in mice BCG immunized as adults compared to mice BCG immunized as infants (Fig 4D). At 8 weeks post-boost the differences in the immune responses found between mice BCG immunized as infants or as adults faded and became comparable (Fig 4E, 4F and 4G). Together these results indicate that respiratory mucosal AdHu5Ag85A boost immunization enhances CD4 and CD8 T cell responses in the lungs of mice BCG immunized as adults to a greater extent than mice immunized as infants.

**Table 1:** Total # of IFN-γ^+^ T cells in BAL in mice boosted at 16 weeks post-BCG (x 10^3^).

	4 wk post-boost		8 wk post-boost	
	Infant	Adult	Infant	Adult
CD4	2.20 +/- 0.49	3.38 +/- 0.90	0.20 +/- 0.06	0.26 +/- 0.02
CD8	0.98 +/- 0.09 *	1.94 +/- 0.23 *	0.19 +/- 0.04	0.37 +/- 0.08

Infant and adult mice were immunized s.c. with BCG, and the AdHu5Ag85A booster vaccine was administered intranasally (i.n.) at 16 weeks post-BCG. The mice were sacrificed 4 or 8 weeks after boosting. Cells isolated from the BAL were stimulated with *M*.*tb* CF + crude BCG, or unstimulated as a control. Cells were stained with extracellular antibodies for T cell markers, followed by intracellular staining for IFN-γ, and analyzed by flow cytometry. Absolute numbers of IFN-γ^+^CD4^+^ and IFN-γ^+^CD8^+^ T cells were calculated (unstimulated subtracted from stimulated). Results are from one experiment per timepoint, n = 4-5/group/timepoint. Data are expressed as Mean ± SEM.

*, p < 0.05.

**Fig 5:**
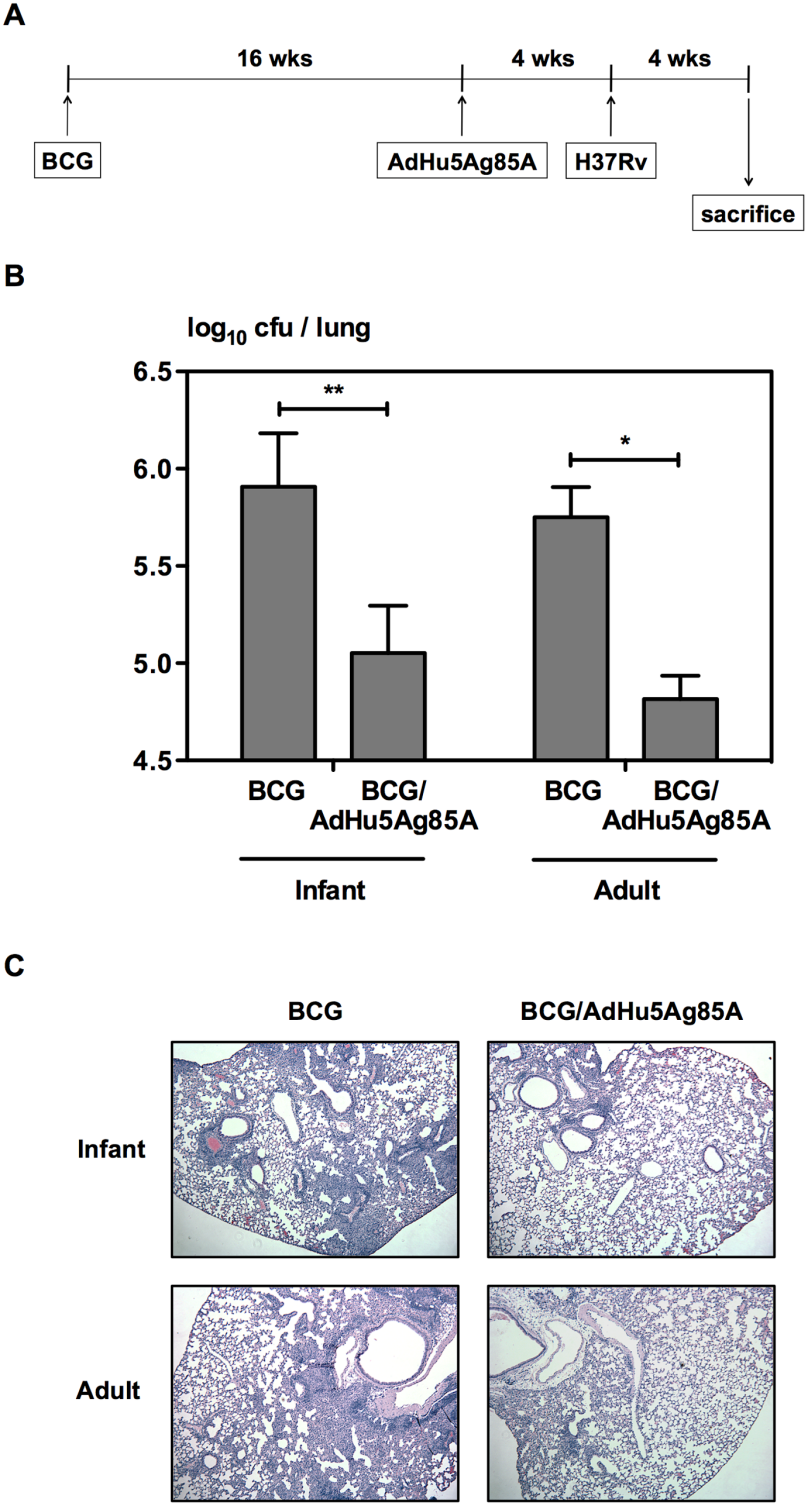
Protection against *M*.*tb* challenge in mice immunized with BCG alone or primed with BCG and boosted with AdHu5Ag85A. Infant and adult mice were BCG immunized, and boosted with AdHu5Ag85A i.n. at 16 weeks post-BCG (BCG/AdHu5Ag85A) (A). The control group was not boosted (BCG). The mice were then challenged i.n. 4 weeks after boosting with *M*.*tb* H37Rv. Bacterial burden in the lungs was assessed at 4 weeks post-challenge by a cfu assay with lung homogenates, expressed as log_10_ cfu per lung (B). Histopathological changes were examined by H&E staining of the lungs (C). Magnification: 5x. Results are from one experiment, n = 5/group. Data are expressed as Mean ± SEM. *, p < 0.05; **, p < 0.005.

To investigate if the observed enhancement in immunogenicity at 16 weeks post-BCG immunization in mice immunized as adults is biologically significant in protecting against virulent *M*.*tb* infection, a group of infant and adult mice were primed with BCG and boosted with AdHu5Ag85A at 16 weeks post-BCG or left without boosting as shown in the experimental schema (Fig 5A). At 4 weeks after boosting mice were challenged with H37Rv and bacterial burden in the lungs as well as histopathology were examined at 4 weeks post-challenge (Fig 5A). Age at BCG priming did not influence the protection capability of BCG against pulmonary *M*.*tb* infection. As expected, respiratory mucosal boosting significantly improved protection against pulmonary tuberculosis in both age groups (Fig 5B). However, levels of protection were comparable in the mice BCG-immunized as infants or as adults, as seen by lung bacterial burden (Fig 5B). Consistent with bacterial control, histopathological changes after infection also did not differ significantly among the groups (Fig 5C). These data suggest that despite increased T cell responses to boosting in mice BCG-immunized as adults, age at BCG immunization did not have an effect on AdHu5Ag85A boost-enhanced protection.

### Longer elapsed time between priming with BCG and AdHu5Ag85A boost immunization leads to a reduced boost effect in mice BCG-immunized as infants but an increased boost effect in mice BCG immunized as adults

**Fig 6:**
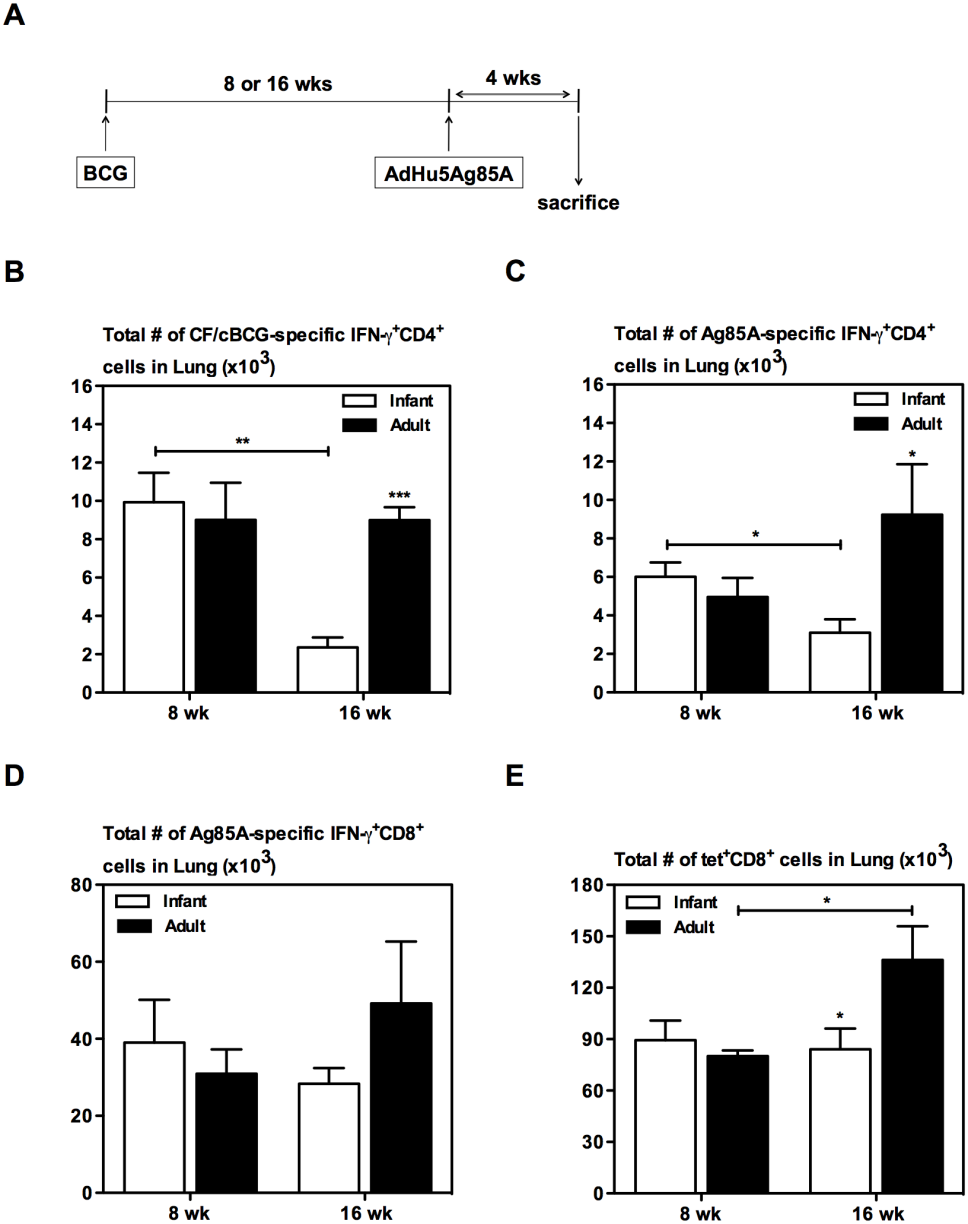
Effect of elapsed time between BCG priming and AdHu5Ag85A boost immunization on Ag-specific responses boosted by AdHu5Ag85A boost immunization. Infant and adult mice were BCG immunized, and boosted with AdHu5Ag85A at 8 or 16 weeks post-BCG (A). The mice were sacrificed 4 weeks after boosting. Cells isolated from the lung were stimulated either with *M*.*tb* CF + crude BCG (B), Ag85A-specific CD4 T cell peptide (C), or CD8 T cell peptide (D), or left unstimulated as a control. Cells were stained and analyzed by flow cytometry. Absolute numbers of IFN-γ^+^CD4^+^ (B, C) and IFN-γ^+^CD8^+^ (D) T cells were calculated (unstimulated subtracted from stimulated). Ag85A CD8 peptide tetramer staining was performed on lung cells, and analyzed by flow cytometry (E). Absolute numbers of tet^+^CD8^+^ T cells were calculated. Results are from one experiment per timepoint, n = 4-5/group/timepoint. Data are expressed as Mean ± SEM. *, p < 0.05; **, p < 0.005; ***, p < 0.0005. All other comparisons (not indicated) were not significant.

An optimal interval between priming and boosting is critical to elicit ideal levels of antigen-specific immune responses. Thus we next analyzed the relation between time elapsed from the BCG priming and AdHu5Ag85A boosting on the activation of Ag-specific T cells. A group of infant and a group of adult mice were immunized with BCG (Fig 6A). At 8 or 16 weeks post-BCG immunization a set of mice immunized as infants or adults were immunized with AdHu5Ag85A (BCG/AdHu5Ag85A) and 4 weeks later antigen-specific responses in the lung were enumerated using flow cytometry (Fig 6A). Lung mononuclear cells were stimulated with either CF+cBCG or Ag85A CD4 or CD8 peptides and analyzed for Ag-specific IFN-γ^+^CD4^+^ and IFN-γ^+^CD8^+^ responses. In addition, lung mononuclear cells were stained for Ag85A CD8 tetramer. In contrast to mice immunized as adults, levels of crude mycobacterial antigen (CF+cBCG) reactive CD4 T cells (Fig 6B) and Ag85A-specific CD4 T cells (Fig 6C) in the lung of mice immunized as infants significantly decreased as elapsed time between BCG priming and AdHu5Ag85A boost immunization was extended from 8 to 16 weeks. On the other hand Ag85A-specific CD8 responses in mice immunized as infants were not affected by increasing the elapsed time between BCG priming and AdHu5Ag85A boosting (Fig 6D). However, extended elapsed time for AdHu5Ag85A boost in mice BCG immunized as adults led to enhanced tet^+^CD8^+^ responses (Fig 6E). Furthermore, delaying the BCG prime immunization from infancy to adulthood resulted in enhanced T cell responses in the lung after AdHu5Ag85A boosting at 16 weeks post-BCG. Together these results indicate that elapsed time between parenteral BCG prime immunization and respiratory mucosal AdHu5Ag85A boost immunization in mice BCG immunized as infants or adults differently affects boost effect of AdHu5Ag85A.

## Discussion

Immaturity of the immune system during early life, with respect to both innate and cellular immune responses is a major challenge for developing vaccines for childhood immunization. Immune maturation in infants is prolonged through 2 years of age resulting in significant differences between the neonatal and adult immune systems [30, 33, 34]. Nevertheless, WHO recommends BCG vaccination to newborns in TB endemic areas due to the increased risk and severity of TB in neonates [35]. Data from human studies that evaluated whether the age at BCG immunization differently impacts activation of antigen-specific T cell responses are inconclusive [19–21]. Importantly, none of these studies investigated whether age at BCG immunization has an impact on protection against pulmonary tuberculosis.

The infant immune system has been shown to be immature with a bias towards Th2 cell polarization and low cytokine production compared with that in adults [17, 18, 22, 24, 25, 30, 36, 37]. However, in the current study, using murine models that reflect the immature immune system in infants and the mature immune system in older children, we showed that infants are able to mount a strong Th1 response equivalent to adults upon BCG immunization (Fig 1), as well as responses with similar multi-cytokine functionality (Fig 2). We also found that BCG-specific responses generated by the immature immune system protect against pulmonary tuberculosis as efficiently as the BCG-specific responses generated by the mature immune system (Fig 5). The equal antigen-specific response in adults and newborns following BCG immunization has also been reported in human studies [16, 22, 38–41]. In contrast to BCG immunization, delaying the administration of tetanus and oral polio vaccines from birth to a few months of age enhances the immune responses [42, 43]. In fact, most vaccines administered during early life generate an inadequate immunity and require more than one dose and adjuvants in the vaccine formulation to establish optimal immunity [16, 44]. Efficacious BCG immunization at birth thus provides evidence that the adjuvant nature of BCG-antigen combinations is able to effectively activate the immature immune system.

The knowledge of the type of memory responses developed in the lung following parenteral BCG immunization in humans is scarce. We showed in the current study that in mice parenteral BCG immunization generates both T_eff_/T_EM_ and T_CM_ in the lung regardless of age at immunization. Interestingly, whereas a significantly higher level of T_CM_ was generated in the lung and spleen when BCG immunization was delayed until maturity of the immune system (Fig 3), levels of T_eff_/T_EM_ in the lung were not affected (Fig 3A). Furthermore, an association between levels of T_eff_/T_EM_ in the lung and protective efficacy, but not between levels of T_CM_ and protective efficacy was observed. Although the T_eff_/T_EM_ population does not expand as much upon secondary infection and is more prone to exhaustion than the T_CM_ population, being present at the site of infection can exert immediate protection, while the T_CM_ population needs time to differentiate into effectors and expand to exert protection [45–47]. Earlier studies have also shown that multifunctional T_EM_ cells generated by BCG immunization in the lung strongly associate with protection [48]. Thus, generating an optimal T_eff_/T_EM_ population in the lung is critical for protection. Implications of these findings in animal models should be extrapolated to humans with caution as immunity at respiratory mucosa in humans is constantly altered due to exposure to different environmental factors.

Many novel candidate tuberculosis vaccines are in the development pipeline to boost BCG-induced immunity and mounting evidence suggests that the benefit of a boost vaccine would be to induce anti-TB immunity in the respiratory mucosa to exert immediate responses upon pathogen entry [49]. However, heterologous novel BCG boost vaccines have advanced in clinical trials without the knowledge of optimal timing for boosting. Boosting BCG with AdHu5Ag85A respiratory mucosal immunization significantly enhances the level of immune protection against *M*.*tb* infection [26, 29]. The current study showed that age at BCG prime immunization does not affect such enhanced protective efficacy of AdHu5Ag85A boosting (Fig 5). However, delaying the BCG prime immunization from infancy to adulthood resulted in enhanced multi-mycobacterial antigen-specific and Ag85A peptide-specific T cell responses in the lung after AdHu5Ag85A boosting at 16 weeks post-BCG (Fig 6). Despite of improved T cell responses both groups of mice were equally protected against pulmonary tuberculosis infection (Fig 5B). This contradicts the traditional view that higher levels of antigen-specific responses following immunization correlate with enhanced immune protection. However, there is indeed evidence of a poor correlation between BCG-induced antigen-specific T cell responses and immune protection against *M*.*tb* infection [50–53]. Furthermore, longer elapsed time (16 weeks as opposed to 8 weeks) between BCG prime immunization and AdHu5Ag85A boosting reduced T cell responses in the lung of mice BCG-immunized as infants (Fig 6). This could be attributed to varying levels of the T_CM_ population in the lung generated by BCG priming, and the declining levels of T_CM_ post-BCG in mice BCG-immunized as infants (Fig 3A). Thus, our data suggest that the ideal time for boosting BCG-induced immunity may vary depending on age at BCG immunization.

In conclusion, our results indicate that the age at parenteral BCG priming has a limited impact on the efficacy of BCG prime-AdHu5Ag85A respiratory mucosal boost immunization-enhanced protection. However, optimal timing for boosting with AdHu5Ag85A to generate sufficient immunogenicity may vary depending on the age at BCG prime immunization. Our findings hold implications for the design of novel TB immunization protocols for humans.
